# Early detection of bacterial pneumonia by characteristic induced odor signatures

**DOI:** 10.1186/s12879-024-10371-7

**Published:** 2024-12-27

**Authors:** Kim Arnold, Alejandro Gómez-Mejia, Miguel de Figueiredo, Julien Boccard, Kapil Dev Singh, Serge Rudaz, Pablo Sinues, Annelies S. Zinkernagel

**Affiliations:** 1 University Children’s Hospital Basel (UKBB), Basel, 4056 Switzerland; 2 Department of Biomedical Engineering, University of Basel, Allschwil, 4123 Switzerland; 3 Department of Infectious Diseases and Hospital Epidemiology, University Hospital Zurich, University of Zürich, Zurich, 8097 Switzerland; 4 School of Pharmaceutical Sciences, University of Geneva, Geneva, 1206 Switzerland

**Keywords:** SESI-HRMS, Exhalomic analysis, Lung infection VOC, Staphylococcus aureus, Streptococcus pneumoniae

## Abstract

**Introduction:**

The ability to detect pathogenic bacteria before the onsets of severe respiratory symptoms and to differentiate bacterial infection allows to improve patient-tailored treatment leading to a significant reduction in illness severity, comorbidity as well as antibiotic resistance. As such, this study refines the application of the non-invasive Secondary Electrospray Ionization-High Resolution Mass Spectrometry (SESI-HRMS) methodology for real-time and early detection of human respiratory bacterial pathogens in the respiratory tract of a mouse infection model.

**Methods:**

A real-time analysis of changes in volatile metabolites excreted by mice undergoing a lung infection by *Staphylococcus aureus* or *Streptococcus pneumoniae* were evaluated using a SESI-HRMS instrument. The infection status was confirmed using classical CFU enumeration and tissue histology. The detected VOCs were analyzed using a pre- and post-processing algorithm along with ANOVA and RASCA statistical evaluation methods.

**Results:**

Characteristic changes in the VOCs emitted from the mice were detected as early as 4–6 h post-inoculation. Additionally, by using each mouse as its own baseline, we mimicked the inherent variation within biological organism and reported significant variations in 25 volatile organic compounds (VOCs) during the course of a lung bacterial infection.

**Conclusion:**

the non-invasive SESI-HRMS enables real-time detection of infection specific VOCs. However, further refinement of this technology is necessary to improve clinical patient management, treatment, and facilitate decisions regarding antibiotic use due to early infection detection.

**Supplementary Information:**

The online version contains supplementary material available at 10.1186/s12879-024-10371-7.

## Introduction

Timely diagnosis of bacterial infections and characterization of bacterial species is crucial for tailored patient treatment, optimal clinical outcome as well as reduction of antimicrobial resistance development [1, 2]. Current diagnostic methodologies for respiratory infections require invasive sampling such as collection of blood and bronchial lavages [3, 4]. In addition, bacterial identification methodologies such as culture in selective media or automated microbial identification systems requiring bacterial growth for identification and antimicrobial susceptibility testing (AST) are regularly deployed, unfortunately requiring days to obtain first results [5–7]. New developments in matrix-assisted laser desorption ionization-time of flight mass spectrometry (MALDI-TOF) have shortened time-to-result and improved diagnostic sensitivity and specificity [8, 9], but still require prior bacterial growth spanning from hours to days [10, 11]. In addition to culture-based methods, molecular methods, including antigen detection of *Streptococcus pneumoniae* and DNA based approaches [12, 13], are much faster than culture-based techniques. However, they cannot differentiate between viable and dead bacteria or between colonization and infection [14–16]. Distinguishing the latter is important because many healthy individuals are colonized with pathogenic bacteria but do not require antibiotic treatment [17, 18]. Colonizing bacteria include non-pathogenic as well as pathogenic bacteria such as for example *S. pneumoniae* and *Staphylococcus aureus* [17, 19, 20]. Colonization by *S. pneumoniae* or *S. aureus* has been shown to be a risk factor for developing serious infections such as bacteremia, endocarditis and pneumonia resulting in increased morbidity, mortality and health care associated costs [21–23].

Because of its high sensitivity and selectivity, mass spectrometry has emerged as a pivotal technology for the identification of bacteria [24, 25]. However, such current mass-spectrometry rely on the collection of bio specimens and subsequent bacterial growth prior analysis. The next generation of mass spectrometric methods aim to bypass such preparatory steps by directly detecting volatile metabolites emitted by proliferating pathogens [26–28]. One such technology is Secondary Electrospray Ionization High-Resolution Mass Spectrometry (SESI-HRMS). It is an advanced and now commercially available analytical technique that enables real-time, non-invasive analysis of volatile metabolites present at trace concentrations in gaseous samples. Its range of applications span from explosives detection [29, 30] to breath analysis [31–33]. Central to this study, it has also shown the potential to detect metabolic activity of in vitro microorganisms, including yeast and bacteria in vitro [32–39] and also in murine infection models [40, 41].

We have previously demonstrated that assessing VOCs from *S. pneumoniae* and *S. aureus* cultures using SESI-HRMS is feasible and not only allows a rapid pathogen detection but also the identification of unique features which could be assigned to specific bacterial species and strains [33].

In this study, we extended this SESI-HRMS method for the early detection of *S. pneumoniae* and *S. aureus* in an advanced in vivo system by using a murine pneumonia infection model. Each mouse served as its own control thanks to the longitudinal monitoring of the whole volatilome emitted through skin and breath by the mouse, before and after infection with the bacterial pathogens. This approach allowed to account for the inherent biological variability between mice resulting in the use of a lower number of mice. A panel of specific VOCs in *S. aureus* or *S. pneumoniae* infection in vivo was identified.

## Materials and methods

### Bacterial strains and growth conditions

*S. aureus* COWAN I (ATCC12598) or JE2 (NARSA *S. aureus*, NR-46543) and *S. pneumoniae* D39::*lux* (parental *Streptococcus pneumoniae* serotype 2, D39 (NCTC 7466) strain, with a chromosomal integrated *luxABCDE* cassette [42, 43] or TIGR4::*lux* (parental *Streptococcus pneumoniae* serotype 4, TIGR4 strain, with a chromosomal integrated *luxABCDE* cassette) [44, 45] (both strains provided by Prof. Sven Hammerschmidt, University of Greifswald) were used in this study. Both the *S. aureus* and *S. pneumoniae* strains were grown axenically from a glycerol stock on a blood agar plate (BA) (Biomerieux) for 14–16 h at 37 °C without or with 5% CO_2_, respectively.

For the growth of *S. aureus* strains, a single colony was used to generate a starting culture in Tryptic-Soy broth (TSB), 37 °C and 220 RPMs for 16–18 h. Following overnight incubation, a fresh batch of TSB media was inoculated with a dilution from the starting culture and incubated at 37 °C, 220 RPMs for 2 h or until bacteria reached exponential phase (optical density of 600 nm (OD600) of 0.6) from which the infection inoculum was prepared for the mice inoculation. For *S. pneumoniae*, a single colony from the starting blood agar plate was streaked onto a fresh Columbia Blood agar (BA) and incubated at 37 °C, 5% CO2 for 10 h. Following this secondary incubation, a fresh batch of Todd-Hewitt broth supplemented with 5% yeast extract was inoculated with enough bacterial cells to achieve a starting OD_600_ of 0.1. This was grown statically in a water bath at 37 °C until an OD_600_ of 0.35–0.4.

### Real-time SESI-HRMS measurements on infected mice

Female 6–10 weeks old C57BL6/j mice (Janvier labs) were kept in individually ventilated cages (IVC) and used for the infection assay. Mice were then transferred to a new and clean IVC cage before mass spectrometric analysis. During measurement, unlimited access to fresh food, water and bedding material was provided. The experimental set-up (Figure S1) used for mice breath measurements consisted of a custom-made airtight plexiglass box (Engel DKV, 4052 Basel, Switzerland; dimensions—width 230 mm, length 500 mm, height 220 mm, volume = 25.3 L) directly connected to an ion source (Super SESI, FIT, Spain) coupled to a high-resolution Orbitrap mass spectrometer (Exactive Plus, Thermo Fisher Scientific, Germany). The cover of the box could be easily removed which facilitated placing of the IVC cage containing the mice. The system was then pervaded with medical-grade air at a flow rate of 0.5 L/min controlled by a mass flow controller. The stream of air entered the airtight box at the opposite side of the SESI-HRMS and was directed via polytetrafluoroethylene (PTFE) tubes into the IVC cage. As the IVC cage was open at the surface, VOCs emitted by the mice were able to escape and were guided towards the SESI-HRMS by directed air flow. VOCs excreted via mice breath/skin were analyzed in real-time for a period of 24 h before infection (baseline) and for 17–24 h during infection. Using an automated switch system (Auto Clicker Typer version 2.0) mass spectra acquisition was alternated every hour between positive and negative ionization mode during the whole measurement period. Mice wellbeing was recorded on a score sheet. Our experimental design included a total of 21 intratracheally infected mice (*n* = 6 for COWAN I, *n* = 5 for JE2, *n* = 6 for D39::*lux* and *n* = 4 for TIGR4::*lux*). The sample size for mouse infections and the different strains of bacteria were calculated using the RStudio (version 2024.04.2) package pwr (version 1.3-0) with an alpha of 0.05, a calculated effect size (cohen d) of 2 and a power of 0.9. We took into consideration the 3Rs principle for reducing the number of mice required for the experiment based on the literature. *Streptococcus pneumoniae* D39::*lux* (serotype 2) and TIGR4::*lux* (serotype 4) both express bioluminescence and their pathogenicity is well studied in mice [43–47]. *Staphylococcus aureus* COWAN I (CC30 or ST30) [48, 49] presents a lower cytotoxicity and virulence compared to the more virulent MRSA strain JE2 (CC8 or ST8) [50].

A 20-µm TaperTip silica capillary emitter (New Objective, U*S. aureus*) and 0.1% solution of formic acid in water were used to generate electrospray in the SESI source. The solvent reservoir pressure was set to 1.3 bar. The sampling line temperature was set to 130 °C and the temperature of the ionization chamber was set to 90 °C. Furthermore, the electrospray voltage was set to 3.5 KV in positive mode and 3 KV in negative mode. Capillary temperature was 250 °C, sheath gas flow rate was set to 50 and S-lens RF level 50.

For mass spectral data acquisition, the Thermo Exactive Plus Tune software (version 2.9) was used in full scan mode with the following parameter settings: Scan range 50–500 *m/z*, polarity negative or positive, microscan number 10, ACG target 10^6^, maximum injection time 50 ms, resolving power 140,000 at *m/z* 200. Internal and external calibration of the mass spectrometer was performed on a regular basis using common background masses [51, 52].

### Intratracheal inoculation of mice with *S. aureus* and *S. pneumoniae*

Following the 24 h baseline measurement, mice weight was recorded and the lung infection was undertaken as follows. Mice intratracheal instillations were performed under anaesthesia using a combination of Ketamine (65 mg/kg) and Xylazine (13 mg/kg) intraperitoneally or by placing the mice in an anesthesia chamber with isoflurane. For intratracheal instillation of either *S. aureus* or *S. pneumoniae*, an inoculum of 1 × 10^7^ CFU/mL of either bacterial strain was prepared in a final volume of 50 µL in sterile PBS. Using an animal-grade intubation laryngoscope (Hugo Sachs Elektronik, HSE) and soft tweezers, 50 µL of bacteria inoculation were applied directly into the trachea of the mice. After the inoculation procedure and once the mice were fully awake, they were placed inside the IVC-cage within the airtight chamber and connected to the SESI-HRMS for real-time headspace analysis during the course of infection. Measurements were conducted for an additional 24 h or until the mice scores showed an increase in distress. Mice were then euthanized with CO_2_.

### Bacterial colony determination/quantification

Bronchoalveolar and nasal lavage (BAL and NAL, respectively) were performed followed by the harvesting of the lungs and nose from infected mice post-mortem. Bacterial numbers were measured in the tissue after processing with 5 mm stainless steel beads (Qiagen) and a tissue lyser II (Qiagen) as well as in the lavage and blood. Spot plating allowed to determine the final number of viable bacterial cells.

### Histology

Mice nose and lung tissues were fixed in buffered formalin and paraffin-embedded. The embedded material was sectioned using a microtome and stained with hematoxylin and eosin (H&E) or Gram staining. H&E was used to visualize inflammation and recruitment of immune cells while Gram staining was used to confirm the presence of either *S. aureus* or *S. pneumoniae*. Tissue embedding in paraffin, slicing and staining was performed at the department for pathology at the University Hospital Zurich. Whole-slide scanning and photomicrography were performed with a Slidescanner Zeiss Axio Scan.Z1 (Zeiss) at the Center for Microscopy and Image Analysis (ZMB) of the University of Zurich. Histology slides were analyzed at the ZMB using the Qpath software v0.4.3 [53].

### Data analysis

#### Data preprocessing

Data preprocessing and statistical analysis was performed with MATLAB (version R2022a, MathWorks, U*SA*). In-house C# console apps based on Thermo Fisher Scientific’s Raw-FileReader (version 5.0.0.38) were used to access raw centroid (intensity cut-off = 10^2^ a.u.) and profile mass spectra. Recalibration of centroid and profile mass spectra was done using reference peaks with formulae which fulfill the “seven golden rules” [54] and common laboratory contaminants [51] which were present in at least 50% of the samples given an initial tolerance of at least 5 ppm. A shape-preserving piecewise cubic interpolation algorithm created in-house was used to assess the experimental error across all *m/z*. In case outliers of reference peaks were detected (i.e., assessed by moving median algorithm), they were excluded from the interpolation process. Afterwards, the centroids and profile peaks were shifted according to the assessed mass error. The procedure was run three times for the whole mass range of all mass spectra to ensure a mass accuracy below 0.5 ppm. Subsequently, kernel density function was applied to bin the histograms of the recalibrated centroids. Following this, an iteration on the bandwidth which controls the smoothness of the probability density curve was performed, in order to ensure Gaussian probability density functions of +/- 1 ppm at full width at half maximum. To generate the data matrix, the centroids present within the aforementioned range were used.

#### Data postprocessing

Data postprocessing was conducted in MATLAB and RStudio (version 2022.07.2). A data matrix of 1,959 time points x 2,814 features in positive mode and a data matrix of 1,958 time points x 4,440 features in negative mode was obtained after data pre-processing. Satellite artefact peaks present in the range of *m/z* 50–62 were excluded from further analysis by only retaining the most intense peak within each *m/z* unit. Hence, a data matrix of 1,959 time points x 2,716 features in positive mode and a data matrix of 1,958 time points x 1,595 features in negative mode resulted. Molecular formula (MF) assignment was performed on protonated and deprotonated species based on accurate mass using elements C, H, N, O and S within a tolerance of 5 ppm and fulfilling the “seven golden rules” [54]. Relative infection time was calculated for each mouse by subtracting the respective inoculation date and time point from all time points of each measurement. Afterwards, signal intensity time traces were computed for all features and smoothed using MATLAB’s moving mean function (directional window length [5 0]). Time of each measurement was then interpolated every hour (-23 h: 1 h: 17 h, excluding 0 h as this was the infection time point; interpolation method pchip) to obtain a common time axis for all mice measurements whereby the shortest measurement among all mice was used to define the last time point (i.e., time point 17 h). Positive and negative matrices were then combined to a matrix containing 40 time points x 4,311 features for each of the 21 mice.

#### Statistical analysis of intratracheal infected mice

The mice data structure corresponded to a multifactorial design of experiments with 2-fixed effects and an interaction. The bacteria strain factor has 4 levels (JE2, COWAN1, D39::*lux*, TIGR4::*lux*). The time factor has 40 levels, including 23 time points before mice infection from − 23 to -1 h, followed by a 17 time points monitoring from 1 to 17 h after infection. To assess the effect of these factors on the acquired longitudinal chemical profiles of all infected mice, dedicated methods like analysis of variance (ANOVA) simultaneous component analysis (SCA) are useful [55]. This method first performs an ANOVA decomposition of the measured data into pure effect matrices, with respect to the factors in the design, before analyzing each matrix with SCA. The mutual orthogonality between effect matrices ensures that each SCA model describes specific effects in the design. However, this property only holds for balanced designs. Alternative ANOVA decomposition strategies have been proposed to handle unbalanced design with ANOVA-SCA, or ASCA. This is the case of general linear models in ASCA+, which yields unbiased estimators of the effects [56], but effect matrices are not mutually orthogonal. Rebalanced ASCA (RASCA) was thus proposed as a solution to also solve for the orthogonality issue [57]. The data were auto scaled before applying RASCA and a custom MATLAB script was used for analysis. Significance of the effects was evaluated using permutation tests [58] where permutations were only allowed within the levels of the other factors for the main effects, but unconstrained for the interaction. RASCA residuals were used to perform Principal Component Analysis to visualize between mice variability. RASCA scores of the time factor were used to evidence effects of infection over time, and the loadings were afterwards used for interpretation and selection of the relevant metabolic features. On the one hand, only the features which contributed most to the highest positive loadings of the component 3 of the time factor, and which showed an increasing abundance after bacterial infection were considered important (only considering the positive loadings of the top 16 features in descending order). On the other hand, also only the features which contributed most to the highest positive loadings of component 2 and showing decreased abundance after bacterial infection were as well considered relevant (only considering the positive loadings of the top 16 features in descending order). This resulted in a total of 16 features showing a decreasing or stable trend after infection and 9 features which show an increasing abundance after infection. For putative compound assignment we used the “lipids and non-lipids main chemical class” metabolite sets in MetaboAnalystR (version 5.0; https://www.metaboanalyst.ca/).

## Results

### Volatile metabolic trajectories during infection

**Fig. 1:**
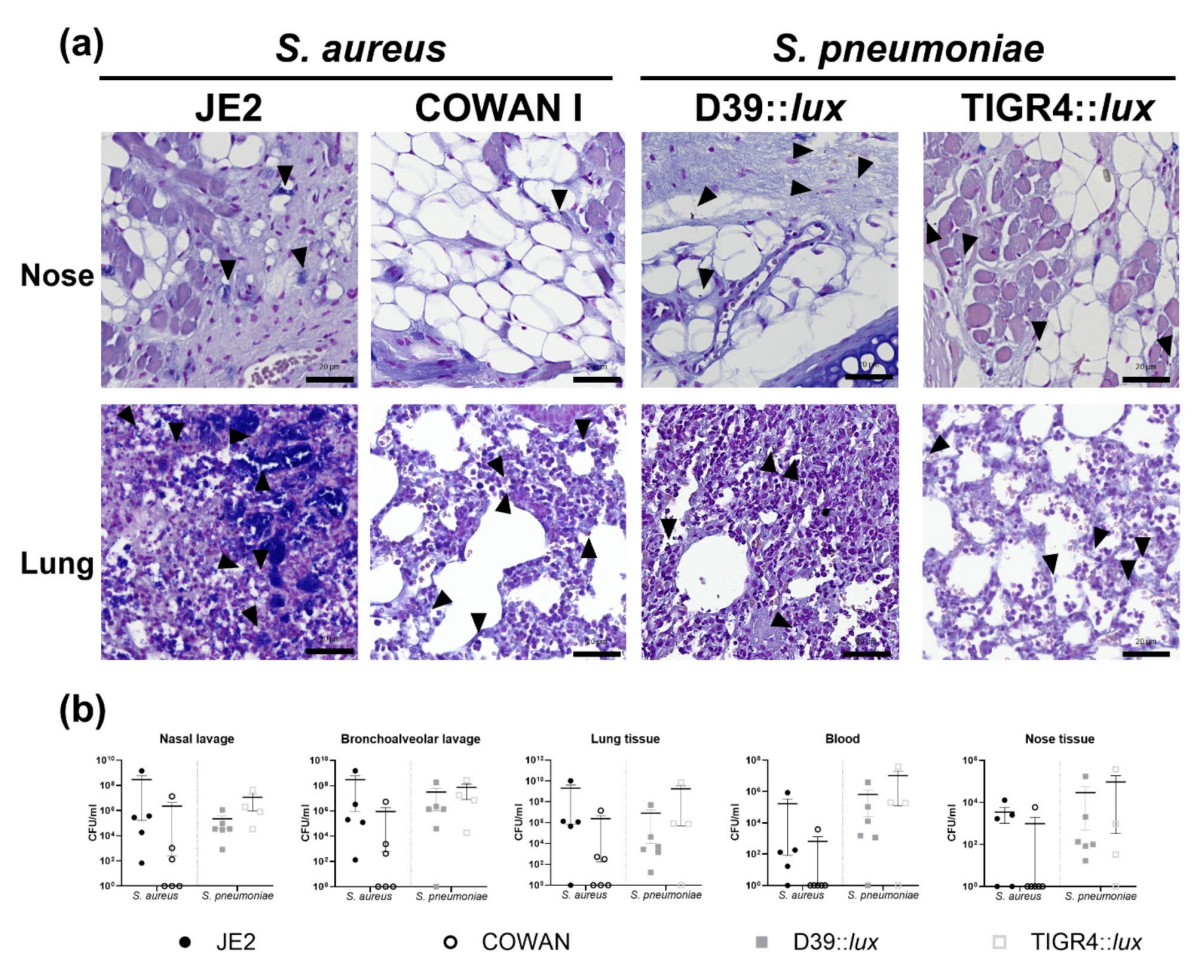
Presence of *S. aureus* and *S. pneumoniae* causing lung infection in mice as confirmed by histology and CFU determination. **(a)** Representative example of lung tissue from mice intratracheally inoculated with different strains of *S. aureus* or *S. pneumoniae* using Gram staining. Black arrows indicate bacteria. The scale bars in images equal 20 μm. **(b)** Quantification of recovered CFUs after processing of lavage and tissue from the lung of infected mice

Histology confirmed bacterial infection and a high infiltration of immune cells and tissue damage in the nose and lungs of intratracheally infected mice as well as the presence of typical *S. aureus* (cocci in clusters) or *S. pneumoniae* (cocci as diplococci or chains) morphologies (Fig. 1a, Figure S2). Histology as well as determination of recovered CFU from lavages, tissue and blood confirmed the presence of bacteria in nose and lungs of infected mice (Fig. 1a and b). Of interest, the recovery of viable bacteria from the upper respiratory tract and blood of the different mice showed a high variability between the mice (Fig. 1b).

**Fig. 2:**
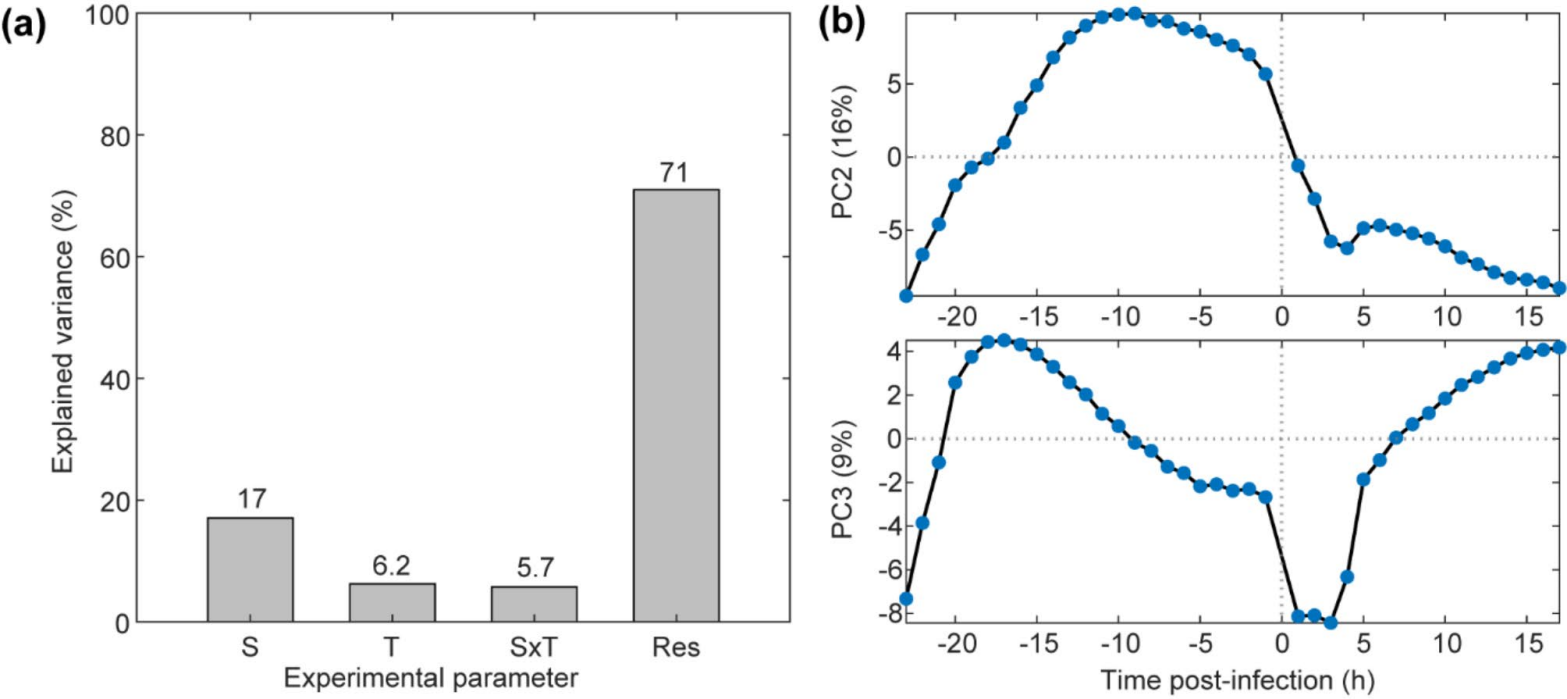
RASCA analysis of intratracheally inoculated mice reveals characteristic bacterial-induced metabolic trajectories during the course of infection. **(a)** Explained variance of the effects included in the experimental design as evaluated with RASCA. A large part of the variance is attributed to the residuals (Res) reflecting the between-mice variability followed by the strain (S) and time (T) main effects, which were both significant as indicated by the permutation test, whereas the strain – time interaction (SxT) was not significant. (**b)** The average scores on the second and third principal components of the time effect are shown. PC2 shows a decreasing trend after the inoculation step whereas PC3 shows an increasing trend

The volatile metabolic fingerprint of intratracheally inoculated mice was successfully monitored over a period of ~ 23 h pre- and ~ 17 h post inoculation in real-time. Data-preprocessing generated a dataset with 2,716 features in positive mode and 1,595 features in negative mode for further investigation. Rebalanced ANOVA Simultaneous Component Analysis (RASCA) [57] was then used for multifactorial modeling involving the *strain* and the *time* factors and their interaction. Following variance decomposition, a proportion of ~ 17% of variance was explained by the first main effect, the bacterial strain (S), ~ 6% by the second main effect, the time (T) and ~ 6% by the strain - time (SxT) interaction. A major part of the variance (~ 71%), was attributed to the residuals (Fig. 2a).

Both strain and time main effects were found to be significant (*p* < 0.001). In order to unveil the latent metabolic trajectories drifting during the course of the infection, the time factor was further investigated. Figure 2b illustrates the scores of the second and third principal components of the time main effect, which showed a characteristic sharp drop during the first four hours after bacterial inoculation. Around five 4–6 h post-inoculation both traces tended to increase again, either to continue this trend over time (PC3) or to slowly decline again (PC2). Figure S3 shows the results of the principal component analysis (PCA) of the residuals, showing the 40 measured time points colored by mouse. The figure highlights some overlap of the colored clusters (i.e., individual mice/convex hulls), but also the high between-mice variability, which explains the largest part of the variance related to the residuals of variance decomposition and also correlates with the high variability in the recovered of viable CFU from the mice at the end of the experiment (Fig. 1b). This approach enables to distinguish the 17% effect associated with the bacterial strain used for the inoculation from the 6% variations related to time of measurement among the inherent inter-individual differences that are expected between biological entities (each mouse). As such, using each mouse as its own pre- inoculation control allowed to reduce the number of mice needed to identify VOCs presenting significant changes over time as well as VOCs that were strain-dependent.

### Departing metabolic trajectories of bacterial lung infection

To identify relevant features which either increase or decrease during the course of bacterial infection in the intratracheally inoculated mice, the features were ranked according to their contribution (loadings) of the components two and three of the time main effect (Fig. 2b). This resulted in a total of 9 and 16 metabolites related signals which showed relevant increasing or decreasing changes over time, respectively, after bacterial inoculation (Table 1).

**Table 1:** – all features showing increased/decreased abundance in infected mice models compared to before inoculation

m/z^a^	Molecular Formula (M)^b^	Adduct	Mass error (ppm)	Compound Hits	Trend post- infection
59.04914	C_3_H_6_O	[M + H]^+^	-0.021	Acetone| Propanal	increase
87.07911	NA	NA	NA	NA	decrease
87.08044	C_5_H_10_O	[M + H]^+^	-0.014	(±)-2-Methylbutanal| 2-Methyl-3-buten-1-ol| 3-Methyl-3-buten-1-ol| Prenol	increase
88.08380	C_5_H_10_O	[M(^13^C) + H]^+^	0.046	(±)-2-Methylbutanal| 2-Methyl-3-buten-1-ol| 3-Methyl-3-buten-1-ol| Prenol	increase
108.04439	C_6_H_5_NO	[M + H]^+^	-0.002	3-Pyridinecarboxaldehyde	decrease
109.06250	NA	NA	NA	NA	increase
122.06814	C_8_H_8_O	[M(^13^C) + H]^+^	-0.050	4-Hydroxystyrene| Phenylacetaldehyde| Acetophenone	decrease
128.02628	NA	NA	NA	NA	increase
128.03531	C_5_H_7_NO_3_	[M-H]^−^	-0.048	N-Acryloylglycine| 4-Oxo-proline| Pyroglutamic acid| Pyrroline hydroxycarboxylic acid| 1-Pyrroline-4-hydroxy-2-carboxylate	decrease
129.05462	C_6_H_8_O_3_	[M + H]^+^	-0.003	2-Dehydropantolactone| (+/-)-Furaneol| 2-Oxo-4E-hexenoic acid	decrease
130.03219	C_5_H_7_NOS	[M + H]^+^	0.613	NA	increase
133.06804	C_6_H_12_OS	[M + H]^+^	-0.923	NA	increase
141.03688	C_7_H_8_OS	[M + H]^+^	0.129	NA	decrease
143.10965	NA	NA	NA	NA	decrease
144.17468	C_9_H_21_N	[M + H]^+^	0.031	NA	decrease
147.10156	C_7_H_14_O_3_	[M + H]^+^	-0.071	2-Hydroxyenanthoic acid| 4-Hydroxyenanthoic acid| 3-Hydroxyisoheptanoic acid	decrease
149.02332	C_8_H_4_O_3_	[M + H]^+^	-0.003	NA	decrease
149.11722	C_7_H_16_O_3_	[M + H]^+^	-0.003	NA	decrease
163.13286	C_8_H_18_O_3_	[M + H]^+^	-0.064	NA	decrease
164.13622	NA	NA	NA	NA	decrease
172.14528	C_8_H_19_N_3_O	[M-H]-	-1.473	NA	decrease
176.13622	C_9_H_18_O_3_	[M(^13^C) + H]^+^	-0.030	4-Hydroxypelargonic acid	increase
191.16416	C_10_H_22_O_3_	[M + H]^+^	-0.055	NA	decrease
218.18318	C_12_H_24_O_3_	[M(^13^C) + H]^+^	0.022	Hydroxydodecanoic acid| 3-Hydroxydodecanoic acid| 12-Hydroxydodecanoic acid| 4-Hydroxylauric acid	decrease
281.24860	C_18_H_34_O_2_	[M-H]^−^	-0.009	Oleic acid| Elaidic acid| Vaccenic acid| cis-Vaccenic acid| Octadecenoic acid |(Z)-13-Octadecenoic acid| 17-Octadecenoic acid|6-Octadecylenic acid	decrease

^a^ Exact mass to charge ratio of the ion

^b^ Molecular formula (M) based on accurate mass only (no isotopic pattern matching performed)

**Fig. 3:**
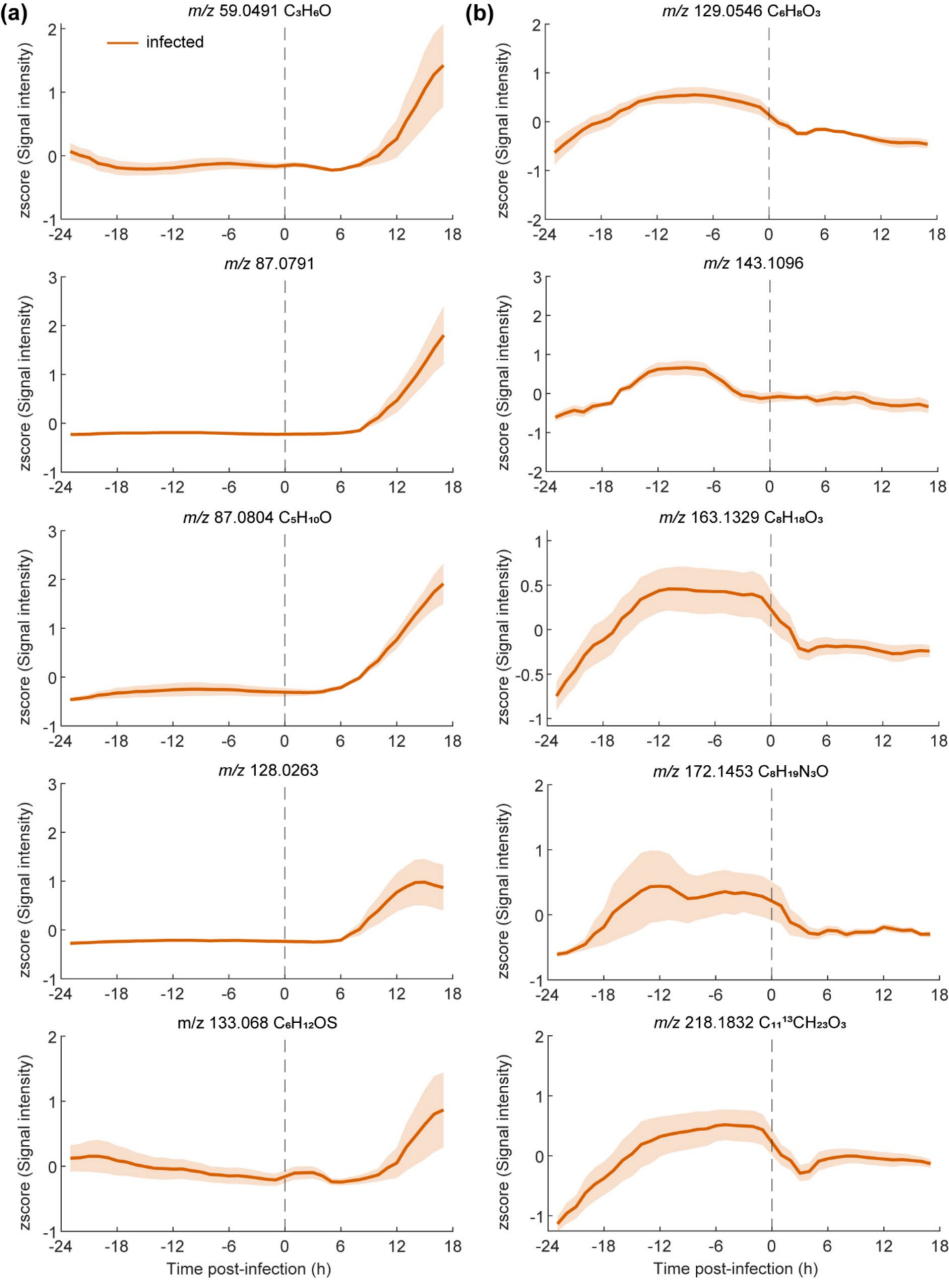
Distinct metabolic trajectories in infected mice. **(a)** Example time traces of five metabolic features contributing most to the increasing time trend in infected mice pre vs. post infection time (dashed line). (**b)** Example time traces of five metabolic features contributing most to the decreasing time trend in infected mice pre vs. post infection time (dashed line). Mean time traces are presented for mice infection (orange line) model along with 95% confidence intervals (light orange areas)

Furthermore, a comparison among the intensity levels of traces before infection vs. after using traces with a strong signal when compared with the uninfected traces (Fig. 3a). Figure 3a shows five examples of features which showed a continuous increase in infected mice (orange) starting between six to eight hours until 17 h post-infection. Figure 3b on the other hand shows five examples of time traces which decreased relatively fast (i.e., within 4 h) after bacterial inoculation in the infected mice. Such downregulation of metabolites suggests that this is likely a host response and probably will not have a predictive value allowing to distinguish one pathogen from another. However, they will likely increase the specificity of an ongoing infection process.

## Discussion

In this study, we used SESI-HRMS to investigate real-time odor evolution during the course of a bacterial infection in vivo in mouse models for bacterial infection using important respiratory pathogens *Staphylococcus aureus* and *Streptococcus pneumoniae* [59–61]. We provide a detailed real-time chemical characterization of the changes of volatile metabolites excreted by infected mice throughout disease progression. We found a panel of VOCs significantly altered within 4–6 h (Fig. 3) after inducing the lung infection. When we evaluated how these metabolic trajectories behave, a distinct pattern was observed for compounds which have been associated with bacterial growth and bacterial infection such as acetone and 2-Methylbuthanal using similar technologies. In addition, we are able to link our findings in terms of m/z with traces at 59, 88 and 141 m/z mass-to-charge ratio in the same studies [40, 62, 63]. The inter-individual variability observed within the mice due to their individual stress response, the fitness state of the bacterial inoculum at the time of infection and how the individual immune system response affected the number of viable bacteria recovered at the end of the experiment in addition to the kinetics and concentration of the traces identified by the SESI. Looking to account for such inter-individual variability, the infection was further confirmed by lung histology and CFU determination of blood, distinct tissues and lavages, however no additional organs were evaluated besides the lungs and nose. Current diagnostic technologies require longer times for the detection of infection agents and thus treatment is often delayed [10, 11, 64, 65]. This works suggests that the detection of VOCs associated to bacterial infection within 4–6 h is amenable with highly sensitive and selective instrumentation, enabling picking up a signal in the very early phases of an infection. The correlation between the observed time traces of the metabolites and disease progression in the infection group suggests an association between these metabolites and the infection process. Whether these markers are produced by the pathogens or are a result of the host response remains to be elucidated and corresponds to an important limitation of this study. Such endeavors will require further chemical characterization of the identified infection-related compounds.

It is also important to state that this study has limitations due to its experimental design and the lack of a non-infected control to account for VOCs changes due to the infection procedure and manipulation. To tackle this, we used each mouse as its own control to account for the major variations expected to occur as is reported within human individuals. However, we cannot rule out inflammatory effects from the procedure itself.

The link between smell and disease has been known since the time of Hippocrates. In the clinical settings, for example, trained dogs with excellent sense of smell detected bacterial proliferation with high sensitivity and specificity [66, 67]. However, the underlying bouquet of volatile metabolites prompting such accurate response remains elusive. So far the technical and regulatory difficulties to introduce less invasive alternatives in the regular diagnostic clinical workflows has prevented the use of odor cues to flag early infectious processes in patients. Studies aiming to use VOCs from pneumonia patients using mass spectrometry have detailed the difficulties in the collection and time to analysis of such samples. Moreover, the patients evaluated in such studies already had advanced disease presenting distinct symptoms of bacterial pneumonia and had been admitted to the intensive care unit due to disease severity [68–70] most likely indicating a late time-point for the detection of bacterial pneumonia.

Here, we aimed for an early detection of bacterial infection in the respiratory tract using real-time monitoring of an in-vivo lung infection model with two important human respiratory pathogens. The ability to continuously monitor the abundance of hundreds of volatile emitted from the skin and breath of mice (i.e. volatilome) during bacterial infection progression and to detect specific traces evolving from the time point of colonization to established lung infection displays the advances and potential of SESI-HRMS.

## Electronic supplementary material

Below is the link to the electronic supplementary material.

Supplementary Material 1

## Data Availability

RAW files obtained from mass spectrometric measurements during the current study are available at MetaboLights, identifier MTBLS11902 (www.ebi.ac.uk/metabolights/MTBLS11902) [71].Additional raw data and datasets used and/or analyzed during the current study are available from the corresponding author on reasonable request.

## Abbreviations

ANOVA: Analysis of variance
AST: Antimicrobial susceptibility testing
BAL: Bronchoalveolar lavage
CFU: Colony forming unit
H&E: Hematoxylin and eosin
IVC: Individually ventilated cages
MALDI-TOF: Matrix-assisted laser desorption ionization-time of flight mass spectrometry
NAL: Nasal lavage
PBS: Phosphate buffer saline
Ppm: Parts per million
RASCA: Rebalanced analysis of variance simultaneous component analysis
SESI-HRMS: Secondary Electrospray Ionization-High Resolution Mass Spectrometry
TSB: Tryptic-Soy broth
VOC: Volatile organic compounds
