# Supplementary material for: Early detection of bacterial pneumonia by characteristic induced odor signatures
Source: BMC Infect Dis. 2024 Dec 27;24:1467. doi: 10.1186/s12879-024-10371-7 (PMC11681641; doi:10.1186/s12879-024-10371-7)
Supplement: Supplementary file 1 — Supplementary Material 1 [file 12879_2024_10371_MOESM1_ESM.docx]

**Early detection of bacterial pneumonia by characteristic induced odor signatures**

Kim Arnold^1,2,*^, Alejandro Gómez-Mejia^3*^, Miguel de Figueiredo^4^, Julien Boccard^4^, Kapil Dev Singh^1,2^, Serge Rudaz^4^, Pablo Sinues^1,2,#^ and Annelies S. Zinkernagel^3,#^

^1^University Children’s Hospital Basel (UKBB), 4056 Basel, Switzerland

^2^Department of Biomedical Engineering, University of Basel, 4123 Allschwil, Switzerland

^3^Department of Infectious Diseases and Hospital Epidemiology, University Hospital Zurich, University of Zürich, 8097 Zurich, Switzerland.

^4^School of Pharmaceutical Sciences, University of Geneva, 1206 Geneva, Switzerland

^*^These authors contributed equally

^#^Correspondence: pablo.sinues@unibas.ch, annelies.zinkernagel@usz.ch

**SUPPLEMENTAL FIGURES**

**
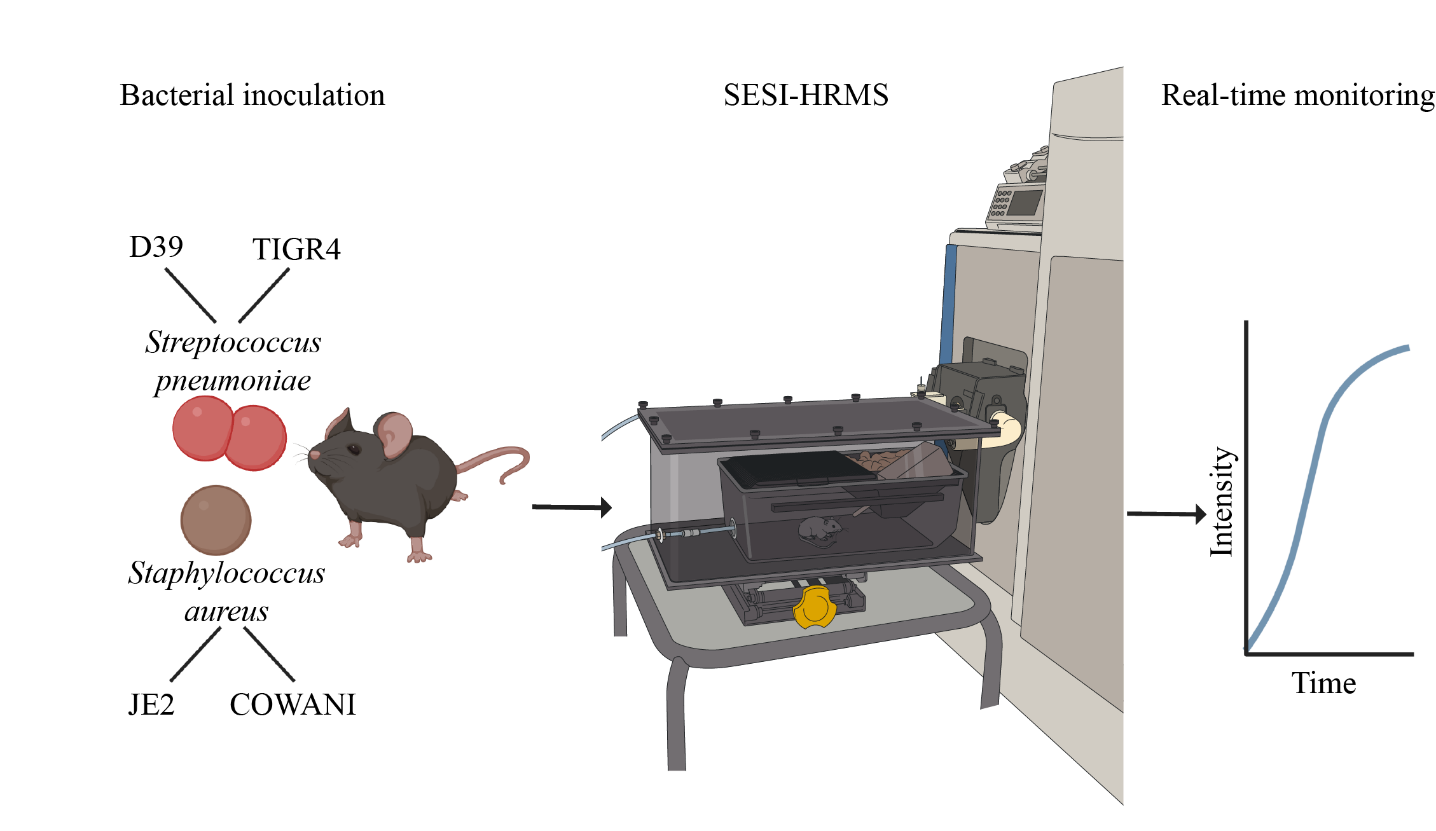
**

**Figure S1.** Schematic illustration of custom-made experimental set-up for the detection of VOCs emitted by mice during bacterial infection. After a 24 h baseline measurement, mice were intratracheal inoculated with a bacterial strain of *S. aureus* or *S. pneumoniae*. Afterwards, mice within an IVC cage were placed inside an airtight plexiglass box coupled to SESI-HRMS. VOCs emitted by mice were then guided towards the SESI-HRMS by a medical-grade air flow of 0.5 L/min, ionized in the SESI and separated according to their mass to charge ratio (*m/z*) in the mass spectrometer (MS), which allows real-time traces of each mass spectral feature to be obtained.

**
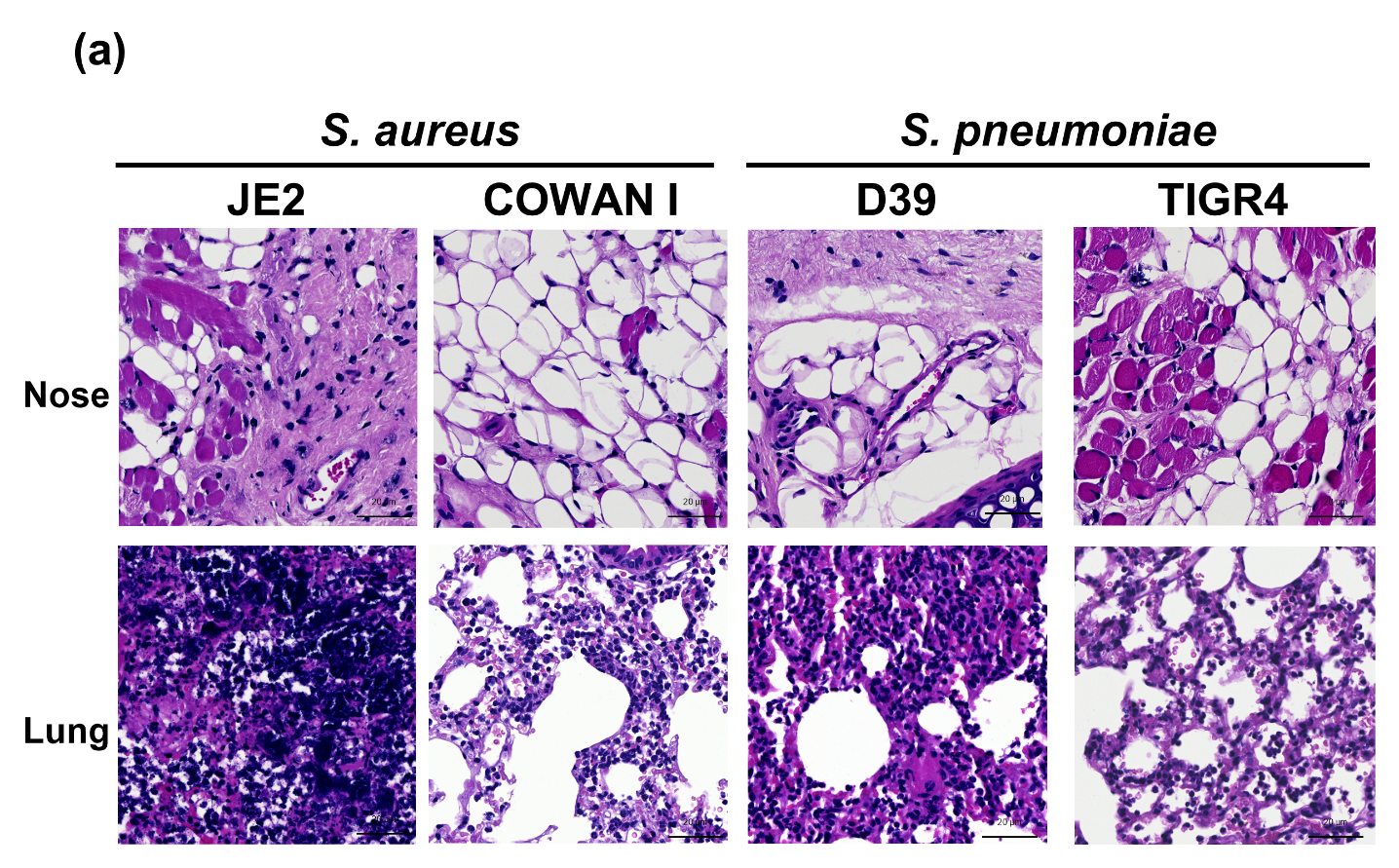
**

**Figure S2. Histology from nose and lungs of mice following intratracheal inoculation with *S. aureus* or *S. pneumoniae*. (a)** Representative examples of nose and lung tissue from mice intratracheally inoculated with different strains of *S. aureus* or *S. pneumoniae* stained with H&E. The scale bars in images equal 20 µm. (Related to figure 1).

**
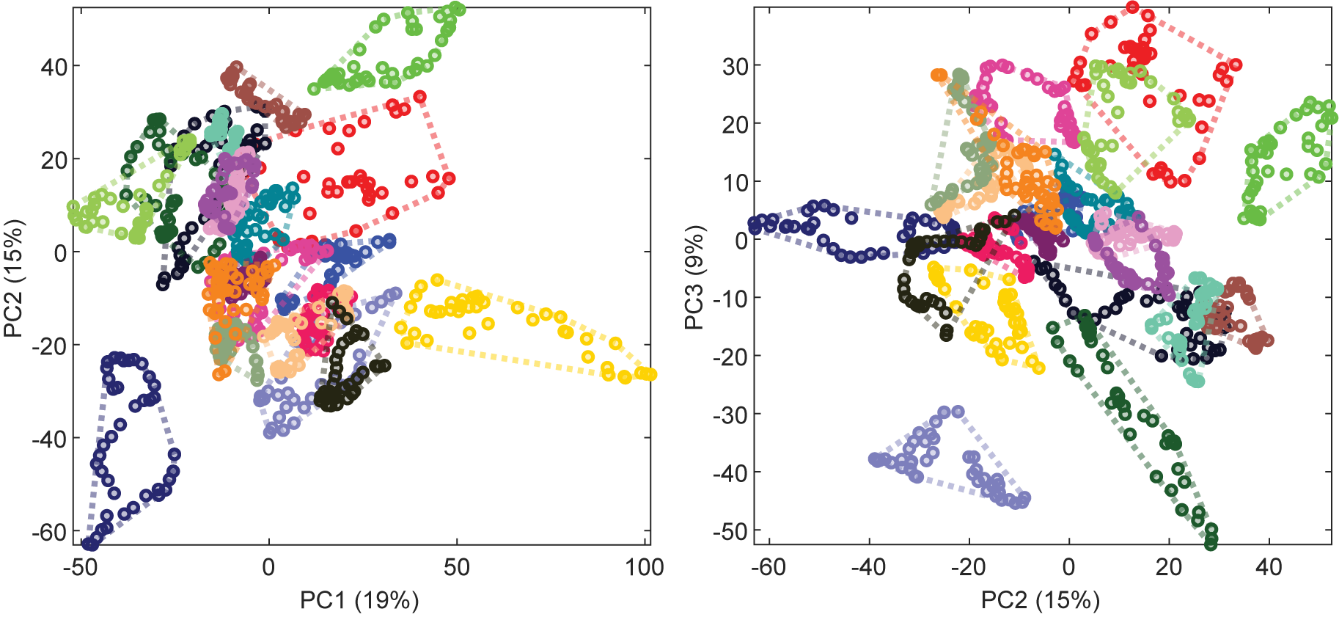
**

**Figure S3. Residual analysis representing mice to mice variability throughout the experiment.**

Scores on principal components associated with the residuals illustrating clearly the between- and within-mice variability (convex hulls), whereby each color represents a mouse and each data point data point indicates one measurement over time. Convex hulls are used to represent mouse boundaries.
